# Intracranial Air Embolism after Inferior Alveolar Nerve Block: A Case Report

**DOI:** 10.5811/cpcem.2020.7.48417

**Published:** 2020-09-13

**Authors:** Megan Gillespie, Chad Gunsolly

**Affiliations:** * Jefferson Health - Northeast, Department of Emergency Medicine, Philadelphia, Pennsylvania; † Jefferson Health - Northeast, Department of Family Medicine, Philadelphia, Pennsylvania; ‡ Jefferson Health - Northeast, Department of Internal Medicine, Philadelphia, Pennsylvania

**Keywords:** inferior alveolar nerve block, intracranial air embolism

## Abstract

**Introduction:**

The number of nontraumatic dental pain emergency department (ED) visits continues to substantially rise in frequency every year. While there are several methods for treating dental pain, an inferior alveolar nerve block (IANB) is a non-narcotic alternative that provides instantaneous relief of severe pain.

**Case Report:**

A 59-year-old male presented to the ED from a dentist’s office for evaluation of a right-sided headache with an associated episode of palpitations and near syncope that developed while receiving an inferior alveolar nerve block. Computed tomography of the patient’s head revealed multiple small foci of air in the right temporalis muscle and in the intracranial venous drainage system. Given the patient’s history of dental procedure, the intravascular introduction of air and local anesthetic was suspected.

**Conclusion:**

Inferior alveolar nerve block procedures can have complications, including hematoma formation, trismus, facial palsy, needle breakage, and in this case, intravascular injection and cerebral air embolism. To perform a successful IANB, it is critical for providers to be familiar with anatomical landmarks and to consistently perform aspiration to confirm that needle placement is not intravascular.

## INTRODUCTION

Approximately two million patients present to the emergency department (ED) with dental complaints annually, which accounts for about 2% of total ED visits.^1^ The inferior alveolar nerve block (IANB) is the most common injection technique used in dentistry and is a non-narcotic option for pain relief in the ED.^2^ An analysis of 10.1 million dental complaint treatment plans from 2010–2013 from the National Ambulatory Medical Care Survey revealed that only 44% of patients received an analgesic while physically being in the ED.^1^ Six percent of dental pain patients received an injection of local anesthetic in the ED, and 35% of dental pain patients received opioid medication.^1^ The number of dental nerve blocks performed is expected to continue to rise as a viable alternative treatment for dental pain during the current opioid epidemic.^1^

## CASE REPORT

A 59-year-old male with a past medical history of hypertension presented to the ED for evaluation of a right-sided headache with an episode of palpitations and near syncope that developed while receiving an IANB for a tooth extraction at a dentist’s office. While his dentist was performing a dental block with lidocaine and epinephrine, he developed acute onset of severe right-sided headache accompanied by palpitations and near syncope with “trouble keeping his eyes open.” The dental extraction procedure was aborted and he was brought to the ED. By the time the patient had arrived to the ED, his palpitations and near syncopal episode had subsided, but his right-sided headache was persistent. The patient denied any other symptoms.

Vital signs at initial presentation included blood pressure of 190/80 millimeters of mercury; heart rate of 73 beats per minute; respiratory rate of 20 breaths per minute; pulse oximetry 96% on room air; and temperature of 98.4° Fahrenheit. Physical exam revealed a middle-aged man in moderate discomfort from pain. His head was normocephalic and atraumatic; no ecchymosis, erythema, or crepitus was noted on his jaw or neck. His pupils were 5 millimeters, equal, round, and reactive to light bilaterally, and without objective ptosis. Cranial nerves II-XII were intact and symmetrical bilaterally. He had 5/5 muscle strength in both the upper and lower extremities bilaterally and 2+ bilateral patella and Achilles deep tendon reflexes. The patient had no ataxia or pronator drift and had a normal finger to nose.

Initial laboratory studies included a basic metabolic panel, complete blood count, coagulation panel, thyroid stimulating hormone level, and troponin. None of these labs demonstrated any significant abnormalities. Twelve-lead electrocardiogram was noted to be normal sinus rhythm with a rate of 63 beats per minute with left axis deviation with high lateral T wave inversion, nonspecific ST changes in anterior leads with no change from previous. A chest radiograph was obtained and was without infiltrates or evidence of cardiomegaly.

**Image 1:**
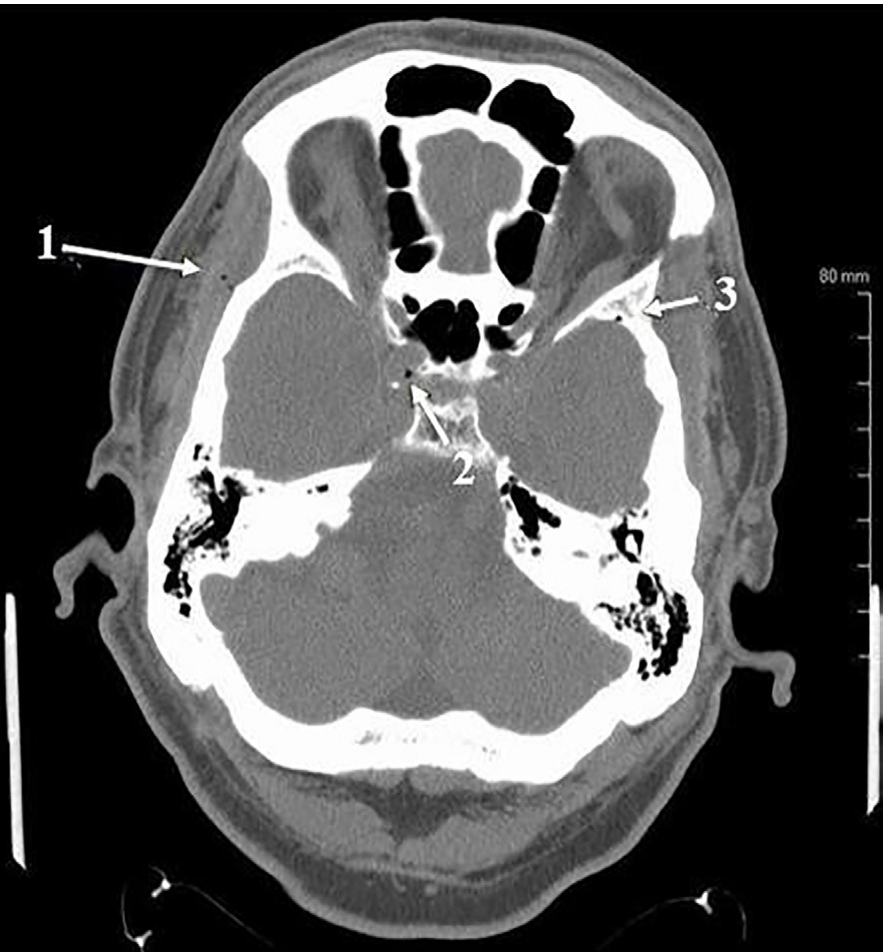
Intracranial air noted on patient’s computed tomography of the head. Arrow 1 indicates multiple foci of air within the right temporalis muscle. Arrow 2 indicates a focus of gas within the cavernous sinus, part of the intracranial venous drainage system. Arrow 3 indicates focus of gas within the sphenoparietal sinus, also part of the intracranial venous drainage system.

**Image 2:**
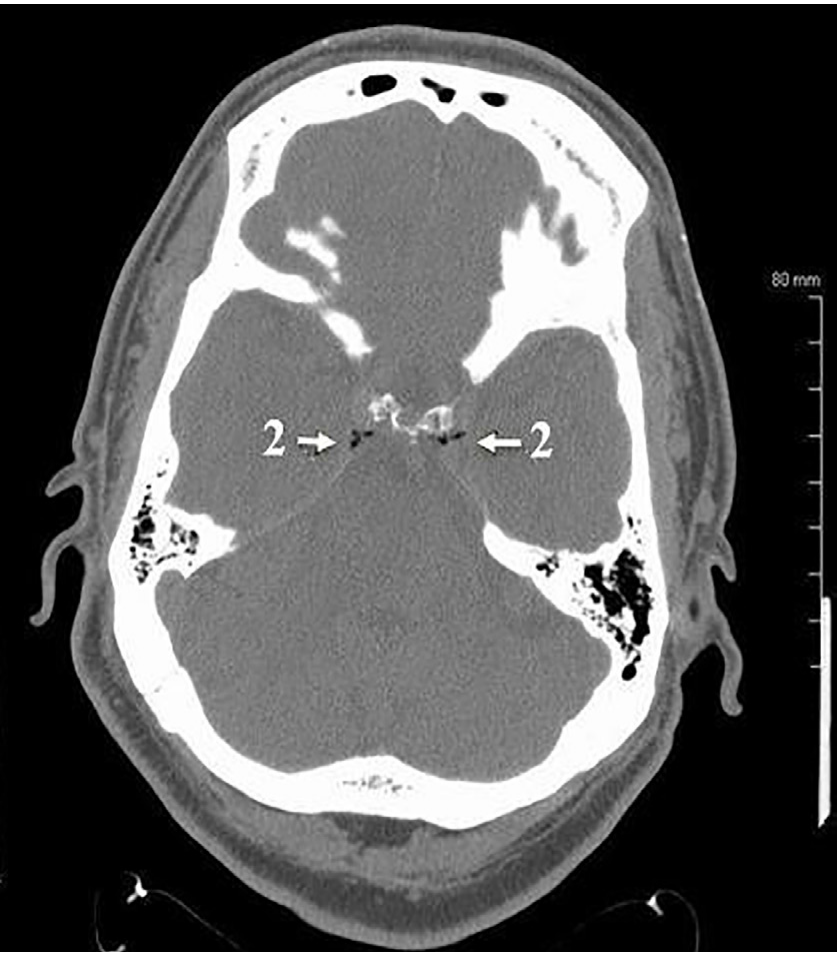
Intracranial air noted on patient’s computed tomography of the head. The arrows marked with 2 indicate multiple foci of gas within the cavernous sinus, part of the intracranial venous drainage system.

Computed tomography (CT) of the head without intravenous (IV) contrast revealed multiple small foci of air predominantly in the expected region of the intracranial venous drainage system (Images 1 and 2). Multiple small foci of air were also noted in the right temporalis muscle. Given the patient’s history of dental procedure the possibility of intravascular introduction of air and local anesthetic was raised (Image 1). There was no evidence of hemorrhage or acute territorial infarction or mass.

Given the findings on the CT head, a dedicated CT neck with IV contrast was performed and showed residual punctate foci of gas in the left transverse sinus and posterior right cavernous sinus. Most of the previously seen bilateral cavernous sinus gas noted on CT of the head was not present on CT neck with IV contrast. The CT neck with IV contrast also reported that the patient was noted to have tortuosity of the extracranial internal carotid arteries with a short segment of the retropharyngeal course at the level of the hypopharynx.

CPC-EM Capsule

What do we already know about this clinical entity?

*Inferior alveolar nerve block (IANB) is a non-narcotic intervention that provides instantaneous relief of severe dental pain*.

What makes this presentation of disease reportable?

*Inferior alveolar nerve block in this case was complicated by intravascular injection of lidocaine with epinephrine and subsequent cerebral air embolism*.

What is the major learning point?

*For successful nerve blocks, be familiar with anatomical landmarks and consistently perform aspiration to confirm needle placement is not intravascular*.

How might this improve emergency medicine practice?

*This case will inform emergency providers of symptoms and management for intracranial air embolism after IANB*.

The patient was admitted to the intensive care unit for continued neurologic and cardiovascular monitoring after discovering the findings of intracranial venous air embolism and air near the carotid sheath on CT from suspected intravascular injection of local anesthetic. While hospitalized, the patient’s headache completely resolved. The patient had a repeat CT of his head and neck 24 hours later with near-total resolution of the previously noted gas in the cavernous sinus region and no acute intracranial infarct or hemorrhage. Neurology evaluated the patient and recommended no additional imaging as repeat CT demonstrated resolution of previously noted venous gas foci, and recommended symptomatic treatment of headache if symptoms recurred. The patient was discharged that next day with completely resolved symptoms.

## DISCUSSION

Experienced providers perform successful IANBs with familiarity of the anatomical landmarks for both the conventional and modified nerve blocking techniques.^2^ The most important clinical landmarks for IANB needle insertion are the coronoid notch and the pterygomandibular raphe.^2^ The needle is inserted into the highly vascular pterygomandibular triangle until bony resistance is felt, and then the needle is minimally withdrawn 1–2 mm. It is then critical to perform aspiration to confirm placement of needle is not intravascular.^2^ Aspirations positive for blood, indicating intravascular placement, are documented to occur about 15% of the time during IANB procedures, the highest frequency of positive aspirations of all intraoral injections.^3^,^4^

The recommended local anesthetic for IANB is 1.8 cubic centimeters of 2% lidocaine with epinephrine +/− bupivacaine for longer duration.^5^ As was the concern for the patient in this case report, one of the potential complications for IANB is the accidental injection of lidocaine with epinephrine into the carotid sheath because of its close anatomical proximity to the pterygomandibular triangle. High-dose or accidental intravascular injection of local anesthetic with vasoconstrictor may result in cardiovascular or central nervous system toxicity with predominant symptoms of hypertension, tachycardia, tachypnea, syncope and/or vertigo, and more infrequent symptoms such as tonic-clonic seizures or diplopia.^5^–^8^

Another potential complication of an IANB is intracranial air embolism. Cerebral arterial gas embolism may cause sudden development of symptoms that range from minor motor weakness and headache to complete disorientation, hemiparesis, convulsions, loss of consciousness, and coma.^9^–^11^ Even small amounts of gas entering the arterial system can occlude functional end arteries.^9^–^11^ Seizures caused by arterial gas emboli typically do not respond to benzodiazepines. Barbiturates can be used instead of benzodiazepines, but ultimately the treatment goal for seizures caused by arterial gas emboli is to reduce the size of the gas emboli. Ways to reduce the size of arterial gas emboli include placing the patient in Trendelenburg or left lateral decubitus position, administering 100% supplemental oxygen, and coordinating initiation of hyperbaric oxygen, which is the first-line treatment of choice.^10^,^12^,^13^ Cerebral venous air emboli have a natural tendency to dissolve in flowing blood, but persistent cerebral venous air embolism can result in stasis and venous infarction.^14^,^15^

## CONCLUSION

Inferior alveolar nerve blocks are a non-narcotic alternative for providing relief to patients who present to the ED with dental pain and are a very common outpatient procedure performed by dentists for anesthesia prior to office procedures. However, IANB procedures can have complications, including hematoma formation, trismus, facial palsy, needle breakage, and in this case, intravascular injection and cerebral air embolism. Frequent review of anatomical landmarks, familiarity with both conventional and modified nerve blocking techniques, and consistent aspiration prior to injection with every IANB procedure will help set the provider up for success in creating analgesia and anesthesia while avoiding complications. Emergency providers should be aware of potential complications from inferior alveolar nerve blocks to allow prompt recognition and proper treatment.
